# New models of care: a liaison psychiatry service for medically unexplained symptoms and frequent attenders in primary care

**DOI:** 10.1192/pb.bp.116.055731

**Published:** 2017-12

**Authors:** Janine Bestall, Najma Siddiqi, Suzanne Heywood-Everett, Charlotte Freeman, Paul Carder, Mick James, Brendan Kennedy, Angela Moulson, Allan House

**Affiliations:** 1 Leeds Institute of Health Sciences; 2 University of York and Hull York Medical School; 3 Bradford District Care NHS Foundation Trust; 4 eMBED Health Consortium, Bradford; 5 NHS Airedale, Wharfedale and Craven Clinical Commissioning Group; 6 NHS Bradford Districts Clinical Commissioning Group

## Abstract

**Aims and method** This paper describes the process of setting up and the early results from a new liaison psychiatry service in primary care for people identified as frequent general practice attenders with long-term conditions or medically unexplained symptoms. Using a rapid evidence synthesis, we identified existing service models, mechanisms to identify and refer patients, and outcomes for the service. Considering this evidence, with local contingencies we defined options and resources. We agreed a model to set up a service in three diverse general practices. An evaluation explored the feasibility of the service and of collecting data for clinical, service and economic outcomes.

**Results** High levels of patient and staff satisfaction, and reductions in the utilisation of primary and secondary healthcare, with associated cost savings are reported.

**Clinical implications** A multidisciplinary liaison psychiatry service integrated in primary care is feasible and may be evaluated using routinely collected data.

Medically unexplained symptoms (MUS) and comorbid physical and mental health conditions place a significant burden on individuals and the economy.^1,2^ Annual costs of MUS have been estimated at £3.1 billion^3^ and of comorbid conditions at £18 billion.^4^ Policy makers suggest innovative approaches be deployed to improve care for patients and make savings across the system.^5^

These patients frequently return to general practitioners (GPs) with unresolved and new complaints. Despite being referred to specialist services their problems remain, which is demoralising for all. Current primary care configurations do not adequately provide for this population. Standard secondary care mental health services rarely have staff with relevant expertise and are insufficiently close to primary care services to influence presentations. Improving Access to Psychological Therapies (IAPT) services are unsuitable due to these patients' high level of medical complexity. Access problems are further compounded by patients not meeting the referral criteria for specialist mental health services.

The skill base in liaison psychiatry services renders them an ideal source of expertise. Until recently such services were hospital-based but they can be integrated within primary care services.^6–8^ We report a novel pilot primary care liaison psychiatry service – the Primary Care Wellbeing Service (PCWS) in Bradford and Airedale, West Yorkshire. The service was targeted to frequent attenders with MUS or psychological difficulties associated with their underlying long-term conditions. This report is a description of the planning, set up and evaluation and feasibility of the preliminary project.

## Method

A partnership board of clinical experts, academics and commissioners met monthly to develop and have oversight of the service; the original impetus came from local Clinical Commissioning Groups (CCGs).

A rapid evidence review helped identify service models, staffing and skill mix, patient selection and outcome measures for the service. Review findings in the context of local practitioner preferences and resources were used to agree the service configuration. The Project Board secured 15 months' funding for the preliminary project. An evaluation by the Commissioning Support Unit (CSU) assessed the feasibility of service design, implementation, collecting the data and effectiveness.

### Data collection

Electronic patient health records (primary and secondary care and mental health) and measures of patient-reported outcomes, experience, and staff experience, were collected as follows:

- Healthcare utilisation: defined as any patient event recorded in the electronic record system; a specific Read code for the service was applied to the patient record at each practice and anonymised data extracted for 12 months prior to and 9 months following referral for secondary care data, and 11 months following referral for primary care and mental health data, providing healthcare activity and costs.
- Clinical effectiveness: at referral and then every 2 months during follow-up using the 9-item Patient Health Questionnaire (PHQ-9),^9^ 15-item Patient Health Questionnaire (PHQ-15),^10^ 7-item Generalised Anxiety Disorder scale (GAD-7)^11^ and the EuroQol 5-dimension, 5-level health status measure (EQ-5D-5L).^12^ A clinician-reported outcome measure assessed perceived improvement in the patient's condition by referring clinician.
- Staff experience: a referrer satisfaction scale, 6 months after the referral or at patient's discharge from the PCWS; and a survey administered at the end of the pilot.
- Patient experience: a brief patient-reported measure at discharge comprising (a) patient satisfaction with the service; (b) two questions asking what was good about the service and what could be improved; and (c) would they recommend the service?
- Case studies were used to illustrate individual formulations and negotiations that took place.

### Data analysis

Feasibility of data collection was judged by the completeness of measures, using summary descriptive statistics. For healthcare utilisation and costs, a before-and-after ‘time series’ approach provided an indication of service effectiveness. Data points were taken from the 12-month period prior to the start of the PCWS and were truncated at 9 months post intervention for secondary care data, giving data points for months 1–21 and 11 months post intervention for primary care and mental health service data at months 1–23. Qualitative data were collated and themes reported.

### The service

The literature review confirmed there was no ‘off the peg’ solution, providing information about key issues to consider when designing the service.

#### Service model and setting

An integrated service with specialist mental health professionals based in and collaborating with three GP practices was established. Practices were selected on the basis of expressions of interest, willingness to commit time and resources. Practices were of average size and served areas of high socioeconomic deprivation. One practice also had a high minority ethnic population.

#### Staffing

The team comprised a team manager, mental health occupational therapist, physiotherapist, psychology assistant, consultant psychiatrist, consultant psychologist, psychologist and administrator. Specialist advice was provided by the consultants. GP practices contributed both GP and practice nurse time.

#### Patient selection and referral

Most studies in the published literature used some combination of case-finding measures for mental disorder alongside frequency or cost of healthcare to identify the target population. A preliminary study in a local practice used the PHQ-9 and a search of GP electronic databases to identify distressed patients and frequent attenders. Of the 100 patients assessed with the PHQ-9, only 6 were identified who were not already in contact with services and who also had significant mood symptoms. They all declined referral. Using standardised case-finding measures failed to identify relevant candidates for this service.

Instead, GPs identified patients using a combination of their own knowledge of patients alongside a risk stratification tool. They focused on those who had a presumed diagnosis of MUS or patients with long-term conditions experiencing significant psychological difficulties, and who were also frequent attenders in primary and secondary care. Attendance was considered to be frequent when a patient had more than the average number of primary care consultations or hospital admissions and when patients' problems remained unresolved and were escalating in cost. A discussion of potential candidates helped achieve consensus about appropriate referrals and practices were then asked to refer ten patients each, providing information on goals for referral and a summary of the patient's health record.

#### Outcomes

Organisational level outcomes (health service use, healthcare costs, medication use) and patient-level outcomes (mental and physical health, physical functioning, and quality of life).

## Results

In total, 28 patients were referred, with 27 appropriate referrals. One was unsuitable due to alcohol dependency and substance misuse. There was a delay of 6 months in receiving referrals from one practice, as the GP lead for the pilot left the practice. Complete data for healthcare utilisation and cost were available for 19/21 patients in 2 practices.

### Health issues

A range of difficulties were identified including neuro-developmental problems; undiagnosed autism; significant health anxieties or preoccupation with illness; chronic pain and overuse of opioids; non-epileptic attacks and medically unexplained loss of movement and pain; and other maladaptive behaviours (e.g. misuse of insulin). Most patients had significant psychosocial difficulties including relationship problems, recent and past life adversity. High levels of physical morbidity such as ischaemic heart disease, chronic obstructive pulmonary disease, arthritis and head injury were also found.

### Patient engagement

Patients were offered an initial joint assessment by two team members; the choice of health professionals took account of referral information about the presentation and degree of readiness to engage. For example, patients reluctant to see a mental health specialist were contacted first by the physiotherapist or occupational therapist. A flexible approach to timing and location of appointments was taken. We were able to engage with 22 of 27 patients either fully or partly with the service.

### Interventions

Initial formulation developed for each patient was reviewed iteratively as alternative interventions were trialled, focused on referral goals. There were four components to interventions as follows:

1. Taking stock and formulating the problem: review of medical notes to reconsider evidence for established diagnoses and medication reviews.
2. Developing a function-based approach, occupationally oriented and focused on improving activities of daily living by accessing community resources; adaptations to home and mobility; introducing non-medical ways of managing pain.
3. Psychological approaches included negotiation of a shared formulation and approach to management, with basic stress and anxiety management. Where indicated, specific therapies such as mindfulness, eye movement desensitisation and reprocessing (EMDR), trauma-focused work and acceptance commitment therapy (ACT).
4. Service-level approaches included non-contingent access to practice staff to manage escalating demands and avoid unscheduled hospital and Accident and Emergency attendance; liaison with GPs and other specialists to agree a consistent approach.

### Feasibility of data collection

Of the 19 patients for whom data could be collected there were only 8 complete data-sets for EQ-5D-5L, GAD-7 and PHQ-9, and 7 complete data-sets for PHQ-15 and EQ-5D-5L.

Healthcare utilisation data from the clinical system were readily available, although it was not possible to collect out-of-hours data. However, gathering information on prescriptions and costs of medication was prohibitively resource intensive.

### Feasibility and effectiveness of the service

**Fig. 1:**
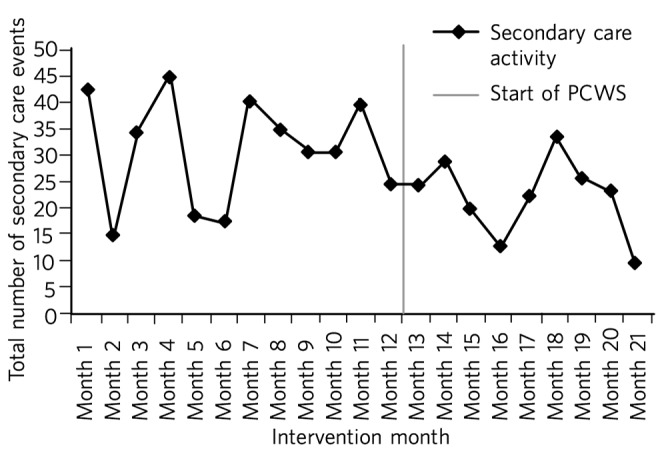
Secondary care activity: time series data. PCWS, Primary Care Wellbeing Service.

Across the whole patient cohort, secondary care activity reduced by an average of nine events per month. In the 9 months after the implementation of the PCWS, 177 fewer events occurred in secondary care (Fig. 1).

**Fig. 2:**
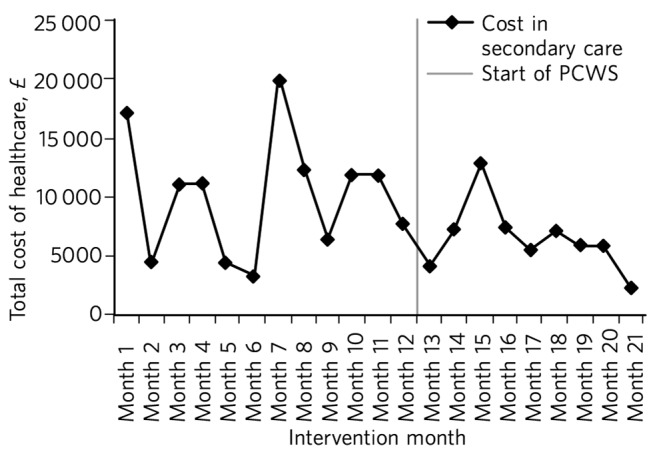
Secondary care costs: time series data. PCWS, Primary Care Wellbeing Service.

Nine months after implementation the total cost of activity was £63 950 less than the previous year (Fig. 1). The cost of secondary care activity reduced by an average of £3702 per month after the implementation of the PCWS (Fig. 2). Primary care activity had also reduced across the whole patient cohort by an average of 11 events per month.

### Primary care

Time spent delivering care reduced by an average of 7 min per month. A substitution effect was observed in primary care with GPs delivering an average of 12 appointments and 127 min less to the patient cohort since the implementation of the PCWS. In contrast, other clinical staff delivered one additional appointment and 120 min more to the patient cohort.

Primary care costs were reduced across the whole scheme by an average of £171 per month for the cohort of 19 patients. In some cases, interventions led to significant changes in symptoms and health-seeking behaviours (Box 1). In others, work is still ongoing and longer-term intervention will be needed.

## Discussion

What works in a research study does not easily translate into routine clinical practice in the National Health Service (NHS). In this study, academics and GP commissioners worked with specialist mental health providers to consider the evidence and identify local resources to design the best service configuration for patients with MUS, long-term conditions and frequent attenders in primary care. There is no short-term solution for these complex patients but a liaison psychiatry service based within primary care and as part of a long-term care plan shows great promise.

### Identifying the patient group for the service and managing complexity

The service presented here addresses a common problem for GPs, which traditional diagnostic categories do not describe well and standard mental health services do not currently manage well. This is not the first attempt at establishing primary care-based liaison services. The service described, however, is unique because it eschews traditional collaborative care approaches^13^ and screening for anxiety and depression widely described in the research literature.^13,14^ In clinical practice that type of screening does not identify the right candidates to work with. The GPs and practice staff identified patients for this study by focusing on those patients with MUS or long-term conditions who were struggling to cope and that were returning with unresolved physical and emotional problems with rising healthcare costs. This was facilitated by a discussion of the case and the development of the vignette prior to referral to the PCWS There was a level of detail in identifying this type of patient that required consideration and scrutiny by practice staff which could not be picked up by electronic systems or case-finding measures alone.

Patients with such complex conditions cannot be managed in standard ways following a protocol. They require a creative approach to person-centred care that supports their identification and management. In almost all cases the GPs were correct in identifying the right patients for the service via their clinical presentation, the exception being someone who required support from specialist addiction services.

**Box 1** Case study

Patient A was a frequent attender at Accident and Emergency, the GP practice and mental health services, with a range of physical and non-psychotic mental health symptoms. She had a suprapubic catheter due to incomplete bladder emptying and detrusor overactivity; she found it too distressing to self-catheterise using a urethral catheter because of a history of sexual abuse. As she was struggling with the suprapubic catheter, an operation was planned to create a conduit between the skin and bladder to make catheterisation easier. However, there was concern that this would not address the underlying reasons for her frequent presentations, and would in fact increase her physical health problems, for example, increasing her risk of urinary infection. Following referral to the Primary Care Wellbeing Service (PCWS), a review of her case notes revealed that her urodynamic studies had been normal. The team liaised with the surgeon, who agreed to a trial of bladder retraining. The team worked to engage patient A and to develop a shared formulation with her about the reasons for her urological difficulties and accept that her physical health difficulties could be managed in a non-operative way. We recognised the importance that all staff conveyed the same message to the patient and that care did not suddenly decrease while other changes in care took place. With input from the practice nurse and the PCWS team, she was able to start passing urine again without the catheter.

### Challenges to service delivery and data collection

Our findings suggest that such services are feasible to deliver but that practices can struggle without sufficient staff to deliver the service. This accords with the literature^15^ which suggests an assessment of practice readiness be performed but this might not account for unexpected changes in practice staffing and infrastructure. Patients and staff that completed satisfaction measures were satisfied with the service, although it is possible that those that did not complete measures did not have such a positive experience. Further, it was not possible to routinely collect self-reported outcome measures in routine practice for this service. Given that these practices were highly motivated to take part, it is unlikely that collecting self-report measures, as part of an evaluation package, would be feasible in less motivated practices. Feedback from staff suggested that they were not able to collect this additional data. Any additional work to use self-report measures in practice needs to consider the burden of additional work for practice staff against the need to collect this information. However, routinely collected data on service use and cost proved to be feasible to collect as this is already part of the existing monitoring systems. Again, out-of-hours information was not collected as part of this. Case studies enabled practitioners to consider how well the patient progressed helping to sustain the service in its early phases using cases as a feedback loop as proposed in the literature.^15^

### Implementing new service models requires a long-term view

There are challenges in setting up and maintaining such services, however. We cannot be sure that they will be cost-effective in the longer term, as the full costs of the service were not examined here only salary costs. This type of analysis would need to be evaluated in a larger study of effectiveness taking account of the commissioning cycle and utilising an economic evaluation. This pilot was only conducted in one metropolitan district in England. Population demographics, health service configurations and commissioning arrangements vary across the UK, and our findings and experience may not be generalisable. Moreover, GP practices taking part were selected for their willingness to engage with the pilot, with one out of the three being unable to launch the proposed service within the project time frame.

Findings from the evaluation are not definitive, but rather provide important data to inform the next stages of service development and evaluation. This pilot demonstrates that service developments can be implemented using NHS resources and commissioning processes, and evaluated using routinely collected data. However, including patient self-report and staff measures, which are not part of usual care, requires additional resources. Administering and collecting paper-based measures for patients and staff proved onerous, with incomplete data collection from all practices despite concerted efforts to collect these by the team.

Questions of sustainability and scaling up need to be considered. In this feasibility study, there were significant reductions in secondary care activity and cost. If such a service could be extended then a further study including full economic costs would be of interest. Such transformation requires commissioners to take a long-term view and to accept that cost savings may be negative or neutral in the first year or more.

## Future proposals

To maintain the momentum and build on this project and other innovative pilots in the UK,^6,7^ we propose setting up a network of interested colleagues to critically consider the future development of primary care liaison psychiatry services. The purpose would be to share experience and to inform further implementation projects and design approaches to the particular problems of scaling up and managing the needs of patients with complex problems who are prone to relapse and likely to require repeated specialist help or longer-term care plans.

## References

[1] Fenton W Stover E Mood disorders: cardiovascular and diabetes comorbidity. Curr Opin Psychiatry 2006; 19: 421–7. 16721175 10.1097/01.yco.0000228765.33356.9f

[2] Naylor C Imison C Addicott R Buck D Goodwin N Harrison T Transforming Our Health Care System: Ten Priorities for Commissioners. The Kings Fund, 2015.

[3] Bermingham S Cohen A Hague J Parsonage M The cost of somatisation among the working-age population in England for the year 2008–2009. Ment Health Fam Med 2010; 7: 71–84. 22477925 PMC2939455

[4] HM Government No Health Without Mental Health: A Cross-Government Mental Health Outcomes Strategy for People of All Ages. Department of Health, 2011.

[5] NHS England The Five Year Forward View for Mental Health. A Report from the Independent Mental Health Taskforce to the NHS in England. NHS England, 2016.

[6] Gathago E Benjamin C Pilot of Enhanced GP Management of Medically Unexplained Symptoms. The Kings Fund, 2014.

[7] Parsonage M Hard E Rock B Managing Patients with Complex Needs: Evaluation of the City and Hackney Primary Care Psychotherapy Consultation Service. Centre for Mental Health, 2014.

[8] van der Feltz-Cornelis CM Van Os TWDP Van Marwijk HWJ Leentjans AFG Effect of psychiatric consultation models in primary care. A systematic review and meta-analysis of randomized clinical trials. J Psychosom Res 2010; 68: 521–33. 20488268 10.1016/j.jpsychores.2009.10.012

[9] Kroenke K Spitzer RL The PHQ-9: a new depression diagnostic and severity measure. Psychiatry Ann 2002; 32: 509–15.

[10] Kroenke K Spitzer RL Williams JB The PHQ-15: validity of a new measure for evaluating the severity of somatic symptoms. Psychosom Med 2002; 64: 258–66. 11914441 10.1097/00006842-200203000-00008

[11] Spitzer RL Kroenke K Williams JB Löwe B A brief measure for assessing generalized anxiety disorder: the GAD-7. Arch Intern Med 2006; 166: 1092–7. 16717171 10.1001/archinte.166.10.1092

[12] Herdman M Gudex C Lloyd A Janssen M Kind P Parkin D Development and preliminary testing of the new five-level version of EQ-5D (EQ-5D-5L). Qual Life Res 2011; 20: 1727–36. 21479777 10.1007/s11136-011-9903-x PMC3220807

[13] Atlantis E Fahey P Foster J Collaborative care for comorbid depression and diabetes: a systematic review and meta-analysis. BMJ Open 2014;4: e004706. 10.1136/bmjopen-2013-004706 PMC3987739 24727428

[14] Bower P Gilbody S Richards D Fletcher J Sutton A Collaborative care for depression in primary care. Making sense of a complex intervention: systematic review and meta-regression. Br J Psychiatry 2006; 189: 484–93. 17139031 10.1192/bjp.bp.106.023655

[15] Byng R Norman J Redfern S Jones R Exposing the key functions of a complex intervention for shared care in mental health: case study of a process evaluation. BMC Health Serv Res 2008; 8: 274. 19105823 10.1186/1472-6963-8-274 PMC2627847

